# Green Tea and Pomegranate Extract Administered During Critical Moments of the Production Cycle Improves Blood Antiradical Activity and Alters Cecal Microbial Ecology of Broiler Chickens

**DOI:** 10.3390/ani10050785

**Published:** 2020-04-30

**Authors:** Vera Perricone, Marcello Comi, Carlotta Giromini, Raffaella Rebucci, Alessandro Agazzi, Giovanni Savoini, Valentino Bontempo

**Affiliations:** Department of Health, Animal Science and Food Safety “Carlo Cantoni” (VESPA), Università degli Studi di Milano, Via dell’Università 6, 26900 Lodi, Italy; vera.perricone@unimi.it (V.P.); marcello.comi@unimi.it (M.C.); carlotta.giromini@unimi.it (C.G.); raffaella.rebucci@unimi.it (R.R); alessandro.agazzi@unimi.it (A.A.); giovanni.savoini@unimi.it (G.S.)

**Keywords:** broiler chickens, phytobiotics, green tea, pomegranate, drinking water, antiradical activity, cecal microbiota

## Abstract

**Simple Summary:**

Since the European Union’s (EU) antibiotic ban in 2006, interest in natural feed additives has largely increased. Natural feed additives are used to prevent diseases and promote growth in chickens, supporting animal health and modulating the development of the gut microflora during stressful situations. In the present study, a bioactive compound from plants belonging to the class of phytobiotics was assessed for its effects on production performance, antiradical activity and gut microflora in broiler chickens. The obtained results show how the tested compound is able to exert beneficial effects on the antiradical activity and gut microbial ecology of birds, even though the chickens’ performance was unaffected.

**Abstract:**

Phytobiotics are usually tested in feed and throughout the production cycle. However, it could be beneficial to evaluate their effects when administered only during critical moments, such as changes in feeding phases. The aim of the trial was to investigate the effect of a commercial plant extract (PE; IQV-10-P01, InQpharm Animal Health, Kuala Lumpur, Malaysia) on growth performance, blood antiradical activity and cecal microbiome when administered in drinking water to broiler chickens during the post-hatching phase and at each change of diet. In the experiment, 480 1-day-old male broiler chicks were assigned to two groups in a 50-day trial. Broilers received drinking water (C) or drinking water plus PE (T) at a rate of 2 mL/L on days 0 to 4, 10–11 and 20–21. PE did not affect performance and water intake, while total antiradical activity was improved (*p* < 0.05). A greater abundance of lactic acid bacteria (false discovery rate (FDR) < 0.05) was found in the T group and the result was confirmed at a lower taxonomic level with higher Lactobacillaceae abundance (FDR < 0.05). Our findings suggest that PE administration during critical moments of the production cycle of broiler chickens may exert beneficial effects at a systemic level and on gut microbial ecology.

## 1. Introduction

In 2006, the European Union banned the use of antimicrobial growth promoters in animal nutrition [1]. This decision led to the result that antimicrobials, other than coccidiostats and histomonostats, were no longer allowed as feed additives [2]. As such, antibiotic alternatives designed to maintain productivity and health became the focus of much research [3,4]. At the present moment, different molecules, compounds, bioactive substances, and active principles have been investigated and are still under investigation [5]. Among them, several classes of feed additives are now available, including probiotics [6,7], prebiotics [8,9], organic acids [10,11], and phytobiotics [12]. Although the benefits of such additives have been proven in most cases, there is still a lack of clarity on their effects, as evidenced by some contrasting results in different trials.

Considering the available feed additive classes, the use of phytobiotics in poultry nutrition could represents a valuable tool [13]. Phytobiotics, also known as botanicals, are plant-derived products that are a natural source of bioactive compounds [14]. Supplementation with phytobiotics for broiler chickens has shown beneficial effects on animal production and the quality of animal-derived products [13]. However, their mechanism of action remains to be elucidated, and different hypotheses have been proposed, in which the antioxidant properties seem to play a major role [15]. Phytobiotics in fact are rich in polyphenolic compounds, which can support the antioxidative capacity by counteracting the harmful effects of free radicals generated during stressful situations, finally resulting in improved general health and better performance of the animals [16].

Phytobiotics were also found to be able to modulate gut microflora [14,17] and its development, which plays an important role in production performance and overall health [18]. It is indeed recognized that the first microbial population colonizing the gut could impact an animal’s entire life span [19]. In this view, the chance to modulate the gut microflora in chickens via a nutritional approach is of particular interest, especially during critical moments of their life, such as the post-hatching phase.

Among phytobiotics, green tea and pomegranate extracts have been shown to improve broiler productivity and antioxidant status [20,21,22], as well as modulate the intestinal microflora [20,23]. Green tea (*Camelia sinensis*) has been widely studied in humans and animals due to its numerous biofunctional properties, including antioxidant, antiviral and anticoccidial activity [24,25]. Most of these properties are ascribed to the high levels of polyphenolic compounds, among which catechins are the most represented group [26]. Similarly, pomegranate (*Punica granatum*) also possesses biofunctional properties, such as antioxidant and anti-inflammatory properties, antimicrobial activity and anticancerogenic effects [27]. A recent in vitro study by Jain et al. [28] showed that the simultaneous use of different plant extracts, including green tea and pomegranate, led to a synergistic enhancement of antioxidant activity. However, combined administration of green tea and pomegranate has not yet been tested in animal nutrition. Green tea and pomegranate have also been demonstrated to affect the intestinal microbiota [23,25], promoting beneficial bacteria in the intestinal tract [29,30].

Until now, the majority of in vivo studies in poultry have investigated the effects of administering phytobiotics in the feed and for the entire rearing period, while the effect of their inclusion in drinking water was scarcely investigated. Phytobiotics supplementation in drinking water might represent a valuable way to perform targeted interventions, limited to the critical moments of the production cycle (e.g., limited number of days during post-hatching phase and transitions between feeding phases). This route of administration could sustain the health of animals when it needs to be supported and boosted, rather than being used for the whole rearing period through the feed. This could then be turned into a smaller amount of phytobiotics used per rearing cycle, with economic advantages in terms of production cost.

To the best of our knowledge, at the present moment no literature is available on the addition of pomegranate to drinking water for poultry. Only two studies considered the effects of including green tea in drinking water [31,32], and neither of these accounted for treatment only during specific critical moments of the rearing cycle. In both these trials, in fact, tea supplementation was performed consecutively from 3 to 10 weeks of age [32] or for a total of 42 days [31]. Kaneko et al. [32] outlined linearly reduced growth performance with an increasing concentration of tea extract, while Rowghani et al. [31] reported improved growth performance following supplementation with 3 mL/L of green tea extract.

The aim of the trial was to evaluate the effect of including a commercial plant extract based on green tea leaves and pomegranate rinds in drinking water on the growth performance, antiradical activity and cecal microbial ecology of broiler chickens during specific critical moments of the rearing cycle.

## 2. Materials and Methods

### 2.1. Animals and Housing

The trial was performed at the Animal Production Research and Teaching Centre of the Polo Veterinario, Università degli Studi di Milano (Lodi, Italy), using 1-day-old male broiler chicks (ROSS 308) obtained from a commercial hatchery (Avicola Alimentare Monteverde, Rovato, BS, Italy). At hatching, all chickens were vaccinated against Marek’s disease, Newcastle disease, infectious bronchitis, and coccidiosis. The chickens were housed in floor pens (2.9 m^2^) on new shavings of white wood in two identical climate-controlled rooms. Water and feed were provided ad libitum. Room temperature was 35 °C for the first 3 days, then decreased weekly by 2 °C to a final temperature of 21 °C at the end of the trial. The study period lasted from the day of hatch until day 50. All procedures were reviewed and approved by the Animal Care and Use Committee of the University of Milan (OPBA_92_2016).

### 2.2. Experimental Design

A total of 480 1-day-old ROSS 308 male broiler chickens were randomly allocated to two experimental groups of 12 pens each at a stocking density of 20 birds/pen. Each experimental room housed six randomly distributed pens per treatment, in order to reduce any environmental effects.

All animals received the same diets (Table 1) formulated to meet the nutrient requirements established by the National Research Council (NRC, 1994).

Diets were provided by Agricom International (Pognano, BG, Italy) according to a three-phase feeding program, in crumbled form for starter and grower phases (0–10 and 11–20 days, respectively), and pelleted form for finisher phase (21–50 days). All experimental diets were formulated and manufactured using the same lots of ingredients and without antibiotics or coccidiostats. Collected feed samples were analysed before the beginning of the trial to determine the content of dry matter (method 930.15), crude protein (method 984.13), ether extract (method 920.39A), ash (method 942.05), Ca (method 968.08), and P (method 946.06) following the relevant Association of Official Analytical Chemists methods of analysis [33].

Experimental treatments consisted of including (treated, T) or not including (control, C) a plant extract (PE) in the drinking water at a dosage of 2 mL/L. Treated birds received PE from 0 to 4 days of the trial and on days 10, 11, 20, and 21, corresponding to the beginning of the trial and the start of the second and third feeding phases. PE was included in one graduated tank for each pen to determine water intake during the treatment period.

The PE was composed of green tea leaves (*Camellia sinensis*) and pomegranate rinds (*Punica granatum*) (IQV-10-P01, InQpharm Animal Health, Kuala Lumpur, Malaysia). During the entire trial, water was provided ad libitum via automatic nipple cup drinker, except during the three treatment periods, when it was provided in graduated plastic tanks placed in each pen. During the trial, growth performance was evaluated at the beginning, at each feed change and at the end of the experiment. On day 50, one representative broiler chicken from each pen was selected and sacrificed; dressing percentage was calculated and blood and cecal content were collected for total antiradical activity assay and gene sequencing, respectively.

### 2.3. Growth Performance and Water Intake

Body weight (BW) and feed intake (FI) of the broilers were determined on a pen basis at 0, 10, 20, and 50 days of age. Mortality was recorded daily together with the BW of dead birds to calculate mortality percentage and correct productive performance results. Water intake was determined on a pen basis during PE administration on days 0–4, 10–11 and 20–21 as the difference between offered and residual water. At the end of the trial, one representative animal was selected from each pen based on pen average BW and sacrificed.

Dressing percentage was calculated by dividing eviscerated weight by live weight. Breast muscle was then removed and weighed, and breast muscle yield was calculated as percentage of eviscerated weight.

### 2.4. Total Antiradical Activity

Blood samples were collected from sacrificed broiler chickens on day 50 in 10 mL vacutainer tubes containing EDTA (Venoject^®^, Terumo Europe NV, Leuven, Belgium) and stored at 4 °C for determination of total blood antiradical activity. Blood samples were processed within 3 h of sampling and analysed in the next 24 h after collection by a Kit Radicaux Libres biological test (KRL, Laboratories Spiral, Dijon, France) following the user protocol. The results were expressed as time (in minutes) required to achieve 50% of maximal haemolysis (half-haemolysis time, HT_50_), which references whole blood and red blood cell (RBC) resistance to a standardized free-radical attack generated from the thermal decomposition of a 27 mmol/L solution of 2,2′-azobis (2-amidinopropane) hydrochloride (AAPH) at 37 °C [34,35,36].

### 2.5. Cecal Microbiota

Cecal contents were collected from sacrificed broiler chickens to perform 16S rRNA gene sequencing. Cecal contents were removed and placed into a sterile tube (Sarstedt, Nümbrecht, Germany), snap-frozen in liquid nitrogen and stored at –80 °C. Bacterial DNA was isolated from cecal contents using the Exgene^TM^ Stool DNA mini kit (Geneall Biotechnology Co., Ltd., South Korea) starting with 200 µg samples following the manufacturer’s procedure. The extracted DNA was quantified using Synergy HTX (Biotek, Winooski, VT, USA) with a final concentration ranging from 3–10 ng/uL. Variable regions V3–V4 of the 16S rRNA were amplified by Polymerase Chain Reaction (PCR) with universal primers for prokaryotes [37]. Amplicon sequencing was carried out on an Illumina MiSeq 300PE platform to obtain raw paired-end reads 2 × 300 bp (BMR Genomics, Padova, Italy). The 16S sequencing data were processed and analysed using CLC Genomics Workbench version 12.0 and CLC Microbial Genomics Module version 4.1 (CLC bio, Arhus, Denmark). The paired-end reads were merged into one high-quality representative by default settings of CLC Workbench (mismatch cost = 1, minimum score = 40, gap cost = 4, maximum unaligned end mismatches = 5). The CLC pipeline was used for primer and quality trimming (trim using quality scores = 0.05; trim ambiguous nucleotides: maximum number of ambiguities = 2; discard reads below length = 5). The SILVA reference database [38] was used for sequence alignment, and sequences were binned into operational taxonomic units (OTUs) based on 97% similarity. The OTU table was further filtered by removing OTUs with low abundance (minimum combined abundance = 10), to get a final abundance table for each sample. The phylogenetic tree was constructed using a maximum likelihood phylogeny tool based on multiple sequence alignment of the OTU sequences (100 most abundant OTUs) generated by the multiple sequence comparison by log-expectation (MUSCLE) tool [39] in the workbench. The maximum likelihood phylogeny tool determines the probability of sequences in the tree, using neighbour joining as the construction method and the Jukes–Cantor model as a nucleotide substitution model. The OTU table was used to calculate alpha diversity indices such as Chao1 and Shannon’s indices.

### 2.6. Statistical Analysis

A completely randomized design was used. Growth performance was analysed using Statistical Analysis System software (SAS version 9.4; SAS Institute Inc., Cary, NC, USA) applying a MIXED procedure for repeated measurements and accounting for the effects of treatment, time and treatment × time interaction. Total weight gain (TWG), total feed intake (TFI), total feed conversion ratio (TFCR), water intake, carcass characteristics, and KRL measurements were analysed using one-way analysis of variance (ANOVA) to compare the means of the two groups using the GLM procedure of SAS. Mortality rate was analysed by PROC FREQ of SAS over the trial period.

The pen represented the experimental unit for growth performance parameters, while the broiler represented the experimental unit for carcass characteristics and KRL measurements. All numerical data in tables are presented as least-square means (LSMeans) accompanied by standard error of the mean (SEM) values. Differences between groups were considered statistically significant at *p* < 0.05, whereas a trend for a treatment effect was noted for 0.05 < *p* < 0.10.

To determine diversity shared among communities in the cecal microbiome of the samples, beta diversity (both weighted and unweighted UniFrac) was calculated in the CLC Workbench (CLC bio, Aarhus, Denmark) and significance was measured by permutational multivariate analysis of variance (PERMANOVA). MicrobiomeAnalyst tool [40] was used for further relevant statistical analysis. During the analysis, the OTUs that did not meet the following parameters were removed: minimum number of counts 1, 5% prevalence in the sample and 1% of samples below the standard deviation. Log transformation was used as a normalization method for downstream analysis, which also includes differential abundant analysis at different taxon levels, performed by the metagenomeSeq package (v3.10, Bioconductor) [41]. Differentially abundant taxa were determined at a false discovery rate (FDR) *<* 0.05.

## 3. Results

### 3.1. Growth Performance, Water Intake, Carcass Characteristics and Total Antiradical Activity

Body weight, weight gain, FI, and FCR are shown in Table 2. The administration of PE in drinking water did not affect growing performance of treated broilers during the different rearing phases (*p* > 0.05). In the same way, no significant differences were seen for mortality rate, dressing or breast percentage. Pen water intake was not influenced by the treatment in the first 4 days of hatching (C: 3.42 L vs. T: 3.36 L; *p* = 0.84), and on days 10–11 (C: 5.75 L vs. T: 7.72; *p* = 0.86) and 20–21 (C: 11.53 L vs. T: 11.75 L; *p* = 0.44) of the trial.

The effects of PE on total antiradical activity are shown in Table 3. Including PE in drinking water during critical moments of the broiler’s rearing cycle significantly improved the total antiradical activity, in both whole blood (HT_50_ blood, *p* < 0.01) and RBCs (HT_50_ RBC, *p* < 0.05).

### 3.2. Cecal Microbiota

Sequencing of amplicons resulting from the amplification product of PCR for variable regions V3–V4 of the 16S rRNA by PCR was performed to investigate the treatment effect on cecal microbiome. Details of sequence read and OTU counts are provided in the supporting materials (Figure S1).

No statistical differences (*p* > 0.05) were seen in alpha diversity measured by bias-corrected Chao1 and Shannon’s indices between C and T groups. Similarly, for beta diversity, no statistical differences (*p* > 0.05) were observed in PERMANOVA (unweighted and weighted UniFrac) between the experimental groups.

**Figure 1:**
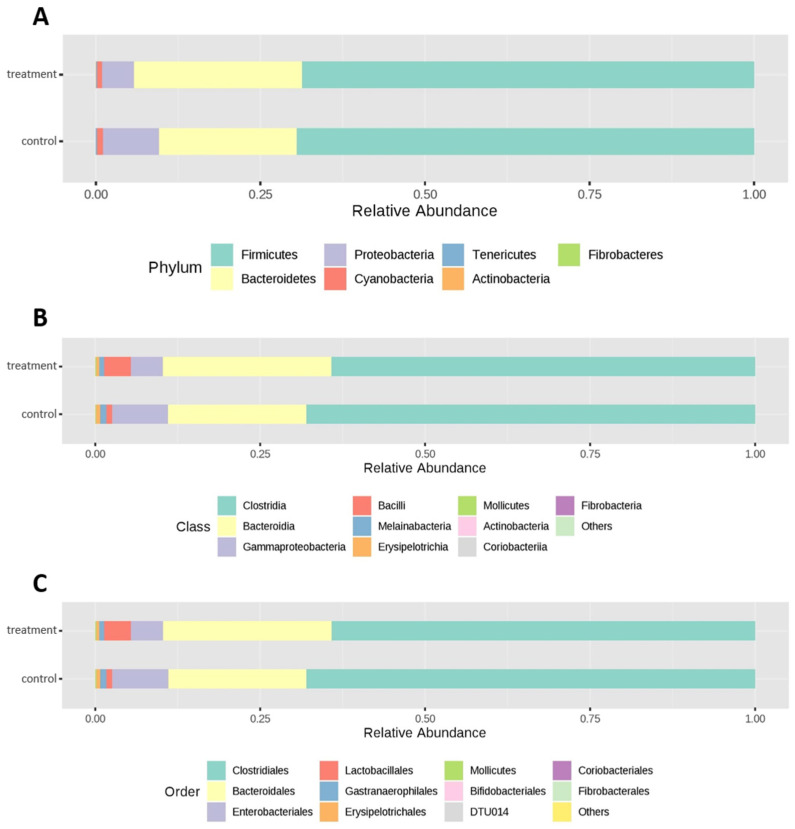
Relative abundance in control and treated groups at different taxon levels: (**A**) phylum, (**B**) class and (**C**) order. Classes and orders with counts <10 are merged and reported as “others”.

Relative abundance at different taxon levels (phylum, order, class) is shown in Figure 1. Firmicutes was found to be the most abundant phylum in both experimental groups, accounting for 69.47% in the C group and 68.65% in the T group. Bacteroidetes emerged as the second most abundant phylum, with 20.94% in the C group and 25.55% in the T group. Proteobacteria were the third phylum, with 8.49% in the C group and 4.84% in the T group. At the class level, Clostridia was the most abundant taxon in both experimental groups, followed by Bacteroidia, Gammaproteobacteria and Bacilli (Figure 1).

Differential abundant analysis was performed to find the significantly different (FDR < 0.05) taxon between the two groups (C and T). No significant differences were found at the phylum level. At the class level, Bacilli were significantly higher in the T group with respect to the C group. Similarly, at the order level, Lactobacillales showed significantly (FDR < 0.05) greater abundance in T animals compared to C animals. At the family level, Lactobacillaceae and Peptococcaceae were significantly more abundant in the T group compared to the C group (FDR < 0.05). Clostridiaceae_1 tended (FDR = 0.06) towards higher abundance in the T group compared to the C group. At the genus level, *Roseburia* was found to be significantly higher in the T group compared to the C group (FDR < 0.05). On the contrary, *Shuttleworthia* was found to be significantly (FDR = 0.04) higher in the C group. *Lactobacillus_ambiguous_taxa*, *Christensenellaceae_R7_ambiguous_taxa* and *Tyzzerella_3* tended (FDR = 0.06) to be higher in the T group compared to the C group. A list of significantly differentially abundant taxa based on *p*-value (*<*0.05) is given in Supplementary Table S1.

## 4. Discussion

Recently, phytobiotics gained increasing attention as a replacement for antimicrobial growth promoters to enhance growth performance and improve animal health [42,43]. The positive effects of phytobiotics have been associated with high polyphenolic content, which can counteract the effect of free radical generation [15], and their ability to modulate gut microflora composition, leading to increased performance [14,22].

In the present study, the lack of positive results as expected might be due to the administration route, the dosage applied, or the duration of supplementation. Generally, supplementation of poultry with green tea and pomegranate extracts was shown to improve broiler productivity [20,21,22]. However, nearly all studies reporting positive effects on growth performance administered the compounds in the feed and for the entire rearing period. To the best of our knowledge, only two studies investigated the single administration of green tea extract to broiler chickens in drinking water, while no data are available on pomegranate or combined supplementation. Rowghani et al. [31] outlined improved growth performance after administration of green tea extract in drinking water at a rate of 3 mL/L, while Kaneko et al. [32] observed a linear reduction of body weight and feed intake with increased concentration of Japanese tea from 6.25 g/L to 25 g/L. In our trial, the lower dosage of green tea and pomegranate mixture was chosen on the basis of the synergistic activity previously evidenced between green tea and other plants, including pomegranate that was able to enhance antioxidant activity in vitro [28]. Finally, the administration of PE for only a few days rather than the total length of the trial could have contributed to the lack of expected results. This is in contrast with the results we obtained in a previous study on post-weaning piglets, with a similar experimental design, which led to an increase in average daily weight gain during the last week of the experimental period [44].

Besides these aspects, a large body of literature highlights the high variability of the efficacy of phytobiotics in improving animal performance and carcass characteristics. This can be explained by the different biological potential of the phytobiotics tested, accounting for the extraction procedure, the part of the plant used, the geographic origin, and the harvest season [45]. According to our findings, Farahat et al. [46] observed no effect on carcass characteristics with different amounts of green tea extract in feed, while Erener et al. [47] and Hamady et al. [20] reported improved carcass characteristics following the administration of green tea and pomegranate extract, respectively.

The administration of PE significantly increased the total antiradical activity of whole blood and RBCs, confirming the beneficial effect of PE in improving antioxidant defences of animals. This result can be attributed to the high polyphenol content of both green tea and pomegranate extract, which is able to prevent reactive oxygen species (ROS) generation and the damage they induce. The proposed mechanism of action for polyphenols is that after being absorbed in the gut, they are bound by blood cells, mainly erythrocytes, leading to enhanced total antioxidant-scavenging capacity of the blood [48]. The antioxidant effects of PE were recently confirmed by Rao et al. [49], who observed reduced lipid peroxidation and increased glutathione peroxidase activity after supplementation with pomegranate peel meal in broiler chickens. Similarly, including green tea extract in the poultry diet increased the glutathione-reduced level in the liver and significantly decreased the malondialdehyde level of meat tissue [46].

The development of intestinal microbiota in poultry plays an important role in production performance and overall health [18], and phytobiotics, including green tea and pomegranate, have been proven to be effective in its modulation [20,23]. It is recognized that colonization of the gut microbiota in critical moments of life could have an impact on an animal’s entire life span [19]. Among the critical moments, the post-hatching phase is one of the most important, since it is when the first gut colonization occurs [50]. Several studies have shown that early gut microflora modulation can affect health and productivity in later stages of a broiler’s life [36,51]. The post-hatching phase, however, is not the only critical moment in defining the gut microbiota composition. The microbial population can also be affected by changes in the diet, with regard to the feed form or its chemical composition [52]. To the best of our knowledge, the present study is the first to investigate the effects of a targeted intervention with phytobiotics at critical moments of the production cycle. Our results show that the administration of PE during the post-hatching phase and changes in the feeding phase did not impact the cecal microbiota composition, keeping the microbial profile in line with the diet used in general practice. The gut microbial population observed in this study was indeed aligned with what was reported by Wei and colleagues [53]. In this review, the authors described the cecal microbial composition of adult birds, reporting Firmicutes as the most abundant phylum, followed by Bacteroidetes and Proteobacteria.

Although the microbial profile was not different between the two experimental groups, relative abundance differences were noted at different taxonomic levels (class, order and family), suggesting a beneficial modulation of gut microflora by PE. In accordance with our findings, Saeed et al. [29] observed higher relative abundance of Bacilli in the ileum and jejunum of broiler chickens following supplementation with L-theanine, an amino acid extracted from green tea [29]. In our study, animals receiving PE showed greater relative abundance of lactic acid bacteria compared to the control group. This result was confirmed at the family level, where Lactobacillaceae and Enterococcaceae were found to be more abundant in T broilers. Also, at the genus level, *Lactobacillus* showed a tendency to be higher in the T group. These findings are of particular interest because lactic acid bacteria are recognized for their beneficial effect in the intestine, regulating the composition of intestinal microflora, developing intestinal immunity and promoting gut health [54]. Lactobacilli can indeed protect against the colonization of pathogenic bacteria through the acidification of the lumen and the production of bacteriocins [55,56].

Besides modulating lactic acid bacteria, PE supplementation also determined some differences at the genus level. *Roseburia_ambiguous_taxa* was found to be significantly higher in animals receiving PE. *Roseburia* genus is a commensal saccharolytic bacteria that produces SCFAs and has been proposed in human medicine as probiotic for restoration of beneficial flora [57]. In addition, a lower abundance of *Shuttleworthia* was observed in the T group. Information about this genus is limited, but a study reported that enrichment of *Shuttleworthia* in the ceca of male broiler chickens was associated with high body weight [58], which was not evidenced in our study.

## 5. Conclusions

The administration of PE in drinking water during the post-hatching phase and at changes between feeding phases can improve total blood antiradical activity and may positively affect the gut microbial ecology of adult broiler chickens by increasing the relative abundance of lactic acid bacteria, with no effect on performance parameters.

## Figures and Tables

**Table 1:** Feed ingredients and nutrient composition of basal diets (as-fed basis).

Ingredients (g/kg feed)	Starter Diet	Grower Diet	Finisher Diet
	0–10 days	11–20 days	21–50 days
Corn	550.5	574.0	616.7
Soybean meal (48% crude protein)	373.0	341.0	292.0
Soybean oil	30.0	43.0	53.0
Dicalcium phosphate	25.0	25.0	21.0
Calcium carbonate	7.0	4.5	5.0
Mineral + vitamin premix ^†^	5.0	5.0	5.0
Sodium chloride (NaCl)	4.0	4.0	4.0
_DL_-Methionine (_DL_-Met)	3.2	1.8	1.6
_L_-Lysine-HCl (_L_-Lys-HCl)	2.3	1.7	1.7
**Nutrient values of diets, analysed (g/kg)**			
Dry matter (g)	877.7	878.2	878.0
Crude protein (g)	229.7	215.1	195.0
Ether extract (g)	56.3	69.4	79.8
Ash (g)	68.2	64.04	58.6
Calcium (Ca; g)	10.0	9.1	8.1
Phosphorus (P; g)	8.7	8.5	7.6
**Nutrient values of diets, calculated (g/kg)**			
Metabolizable energy (kcal/kg)	3002.5	3099.9	3200.1
Lysine (Lys)	10.0	8.3	7.6
Metionine + cysteine (Met + Cys)	6.4	4.9	4.4

^†^ Provided the following per kg of diet: vitamin A, 11,250 IU; vitamin D_3_, 5000 IU; vitamin E, 60 mg; MnSO_4_·1H_2_O, 308 mg; ZnSO_4_·1H_2_O, 246 mg; FeSO_4_·1H_2_O, 136 mg; CuSO_4_·5H_2_O, 39 mg; KI, 2.4 mg; Na_2_SeO_3_, 657 μg; 6-phytase EC 3.1.3.26, 750 FTU; endo-1, 4-beta-xylanase EC 3.2.1.8, 2250 U.

**Table 2:** Effects of plant extract supplementation on growth performance parameters and carcass characteristics of broilers. Data shown as LSMeans ± SEM.

Item	Groups		SEM	*p-*Value		
	C	T		Treatment	Time	Treatment × Time
No. Birds/Pen	20	20				
BW (kg/pen) ^1^						
0 day	0.883	0.872	0.842	0.469	<0.001	0.638
10 day	6.195	6.215				
20 day	18.312	18.332				
50 day	74.892	73.106				
Gain (kg/pen) ^1^						
0–10 days	5.312	5.342	1.075	0.445	<0.001	0.533
11–20 days	12.117	12.117				
21–49 days	56.580	54.774				
TWG	74.008	72.233	1.667	0.460		
FI (kg/pen) ^1^						
0–10 days	6.393	6.343	0.819	0.276	<0.001	0.294
11–20 days	18.102	18.158				
21–49 days	122.808	120.863				
TFI	147.302	145.363	1.257	0.287		
FCR (kg/kg) ^1^						
0–10 days	1.20	1.19	0.035	0.721	<0.001	0.689
11–20 days	1.50	1.50				
21–49 days	2.18	2.23				
TFCR	2.00	2.02	0.036	0.613		
Mortality (%)	3.33	5.83		0.190		
Carcass characteristics						
No. birds ^2^	12	12				
Dressing (%)	75.59	76.83	0.56	0.133		
Breast (%)	21.41	22.41	0.66	0.293		

Note: *p* < 0.05 considered significantly different, 0.05 < *p* < 0.1 considered tendency. SEM: standard error of the mean; BW: body weight; TWG: total weight gain; FI: feed intake; TFI: total feed intake; FCR: feed conversion ratio; TFCR: total feed conversion rate. C: animals receiving no supplementation; T: animals receiving 2 mL/L green tea and pomegranate extract in drinking water at days 0–4, 10–11 and 20–21. ^1^ Corrected for mortality; mortality and BW of dead birds were recorded daily to calculate mortality percentage and correct productive performance results. ^2^ One representative animal from each pen was selected based on pen average BW.

**Table 3:** Effects of plant extract supplementation on total antioxidant activity. Data shown as LSMeans ± SEM.

	Groups		SEM	*p*-Value
Item	C	T		
No. birds ^1^	12	12		
HT_50_ whole blood, min.	69.17	76.52	4.91	<0.001
HT_50_ RBC, min.	56.72	61.28	3.45	0.023

Note: *p* < 0.05 considered significantly different. HT_50_: time (minutes) required to achieve 50% of maximal haemolysis; RBC: red blood cell. C: animals receiving no supplementation; T: animals receiving 2 mL/L green tea and pomegranate extract in drinking water at days 0–4, 10–11 and 20–21. ^1^ One representative animal from each pen was selected based on pen average BW; blood samples were obtained at slaughter.

## Supplementary Materials

The following are available online at https://www.mdpi.com/2076-2615/10/5/785/s1. Figure S1: (A) Mean number of 16S rRNA sequence reads and (B) OTU counts detected in cecal samples of broilers in treated (T) and control (C) groups. Table S1: Significantly different taxa according to *p*-value (≤0.05) shown by differential abundance analysis between the two experimental groups.
