# Supplementary material for: Green Tea and Pomegranate Extract Administered During Critical Moments of the Production Cycle Improves Blood Antiradical Activity and Alters Cecal Microbial Ecology of Broiler Chickens
Source: Animals (Basel). 2020 Apr 30;10(5):785. doi: 10.3390/ani10050785 (PMC7277556; doi:10.3390/ani10050785)
Supplement: Supplementary file 1 [file animals-10-00785-s001.zip › Figures and supporting materials R1/Table S1.pdf]

Table S1. Significantly different taxon according to P-value ($P \leq 0.05$) shown by differential abundant analysis among the two experimental groups.

TAXON	p-value
**Class**	
Bacilli	<0.01
**Order**	
Lactobacillales	<0.01
**Family**	
Lactobacillaceae	<0.01
Peptococcaceae	<0.01
Clostridiaceae_1	<0.01
Enterococcaceae	0.05
**Genus**	
Roseburia[1]	<0.01
Shuttleworthia	<0.01
Tyzzerella 3	<0.01
Lactobacillus[1]	<0.01
Christensenellaceae R7 [1]	<0.01
Anaerostipes	<0.01
Harryflintia	<0.01
Clostridium sensu stricto1	<0.01
Ruminococcaceae UCG014 [1]	0.01
Butyricicoccus	0.01
Ruminococcus gauvreauii group [1]	0.02
Lachnospiraceae UCG006	0.03
Anaerotruncus [1]	0.03
Merdibacter	0.04
Blautia [1]	0.04
Enterococcus [1]	0.04
Ruminiclostridium 5	0.04
Candidatus Arthromitus	0.05
**Species**	
Gut metagenome	<0.01
Merdibacter massiliensis	0.04
uncultured Clostridiaceae	0.05

Bolded taxon have showed a significant difference at FDR ≤ 0.05
[1]Ambiguous taxa
