# Supplementary material for: Green Tea and Pomegranate Extract Administered During Critical Moments of the Production Cycle Improves Blood Antiradical Activity and Alters Cecal Microbial Ecology of Broiler Chickens
Source: Animals (Basel). 2020 Apr 30;10(5):785. doi: 10.3390/ani10050785 (PMC7277556; doi:10.3390/ani10050785)

Figure S1. Mean number of 16S rRNA sequence reads (A) and the number of OTU counts (B) detected in the cecal samples of broilers in treated (T) and control (C) groups.

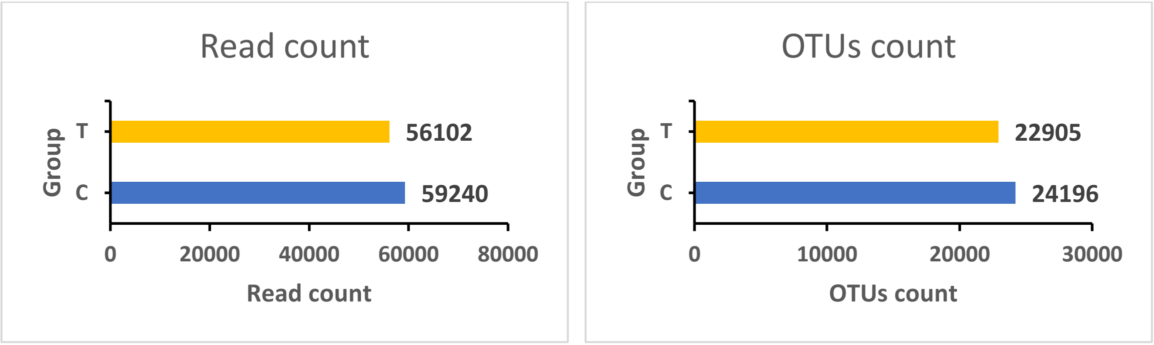

Supplement: Supplementary file 1 [file animals-10-00785-s001.zip › Figures and supporting materials R1/Figure S1.pdf]
